# Preparation, Characterization and Manipulation of Conjugates between Gold Nanoparticles and DNA

**DOI:** 10.3390/nano6090167

**Published:** 2016-09-08

**Authors:** Gennady Eidelshtein, Moran Fattal, Gavriel Avishai, Benjamin Kempinski, Clelia Giannini, Alexander Kotlyar

**Affiliations:** 1 Department of Biochemistry and Molecular Biology, George S. Wise Faculty of Life Sciences and the Center of Nanoscience and Nanotechnology, Tel Aviv University, Ramat Aviv, Tel Aviv 69978, Israel; g.eidelshtein@gmail.com (G.E.); moranfattal@gmail.com (M.F.); gabihem@walla.co.il (G.A.); kempinski@mail.tau.ac.il (B.K.); 2 Department of Chemistry, University of Milan, via Golgi 19, 20133 Milan, Italy; clelia.giannini@unimi.it

**Keywords:** atomic force microscopy, gold nanoparticles, DNA nanotechnology, poly(dG)-poly(dC)

## Abstract

Here we described the preparation and characterization by atomic force microscopy of dumbbell-shaped conjugates between 450 bp double-stranded DNA polymer, poly(dG)-poly(dC), and 5 nm gold nanoparticles (GNPs). We have demonstrated that the size of the nanoparticles in the conjugates can be increased in a controlled fashion. Application of the conjugates for measuring the electrical conductivity of DNA is discussed.

## 1. Introduction

It was demonstrated in 1996 by Mirkin [1] and Alivisatos [2] that conjugates of gold nanoparticles (GNPs) with DNA are self-assembled into complex structures. Since then, many studies have demonstrated the formation of various two- and three-dimensional DNA-nanoparticle structures and arrays [3,4,5,6,7,8,9,10,11,12]. These structures exhibit unique optical and self-assembling properties and can be used for the production of novel nanoscale electrical and optical devices, providing new solutions to many challenges in the fields of nanobiotechnology, nanomedicine and material sciences. In majority of works on the DNA-nanoparticle conjugates published so far, relatively short (up to 100 bases) single-stranded oligonucleotides were attached to the nanoparticle by thiol chemistry. Annealing of complementary sequences linked to different nanoparticles yielded structures bearing different numbers of nanoparticles connected to each other by ds DNA bridges [3,4,6,7,11,12]. In our earlier work [13], we synthesized conjugates of GNPs with long (hundreds of base pairs) ds poly(dG)-poly(dC). The DNA molecules were linked to the nanoparticles via a thiol moiety introduced at the end of the G-strand composing the DNA and the conjugates bearing different numbers of nucleic acid molecules per particle were separated by electrophoresis. The formation of dumbbell-shaped conjugates between long (hundreds of base pairs) ds poly(dG)-poly(dC) molecules and GNPs has never been demonstrated before. Due to its regular structure, DNA composed of G- and C-homopolymers provides better conditions for π overlap compared to random sequence DNA. In addition, guanines, having the lowest ionization potential among DNA bases [14], promote charge migration through the nucleic acid [15]. These properties make poly(dG)-poly(dC) a potential candidate to create nanoelectrical devices and circuits. 

Direct measurements of DNA conductivity are very challenging and require a good electrical connection between the nucleic acid and the metal electrode. Gold nanoparticles, if placed at both ends of the DNA, can strongly accelerate the efficiency of electron transfer between the electrode and the nucleic acid, thus making it possible to conduct direct electrical measurements on single DNA molecules. 

Here we report the synthesis of nanodumbbells composed of two 5 nm GNPs and a 450 bp poly(dG)-poly(dC) DNA. These dumbbell-shaped conjugates are characterized by electrophoresis and atomic force microscopy (AFM). We also demonstrate that the nanoparticles in the conjugate can be enlarged in a controlled fashion. The enhancement method described here can be used for increasing the diameter of GNPs attached to various DNA-based architectures, such as, for example, complexes of DNA origami with nanoparticles [8].

## 2. Results and Discussion

### 2.1. Synthesis, Purification and Atomic Force Microscopy (AFM) Characterization of Dumbbell-Shaped DNA–Gold Nanoparticle (GNP) Conjugates

The synthesis of the DNA-GNP conjugates includes the following stages: 1. Enzymatic extension of SH-poly(dG)-poly(dC)-SH, a ds DNA functionalized with thiols at both ends of the nucleic acid for conjugation with GNPs; 2. Incubation of the DNA with GNPs; 3. Separation of the conjugates formed during the incubation from the excess of nanoparticles. 

**Figure 1:**
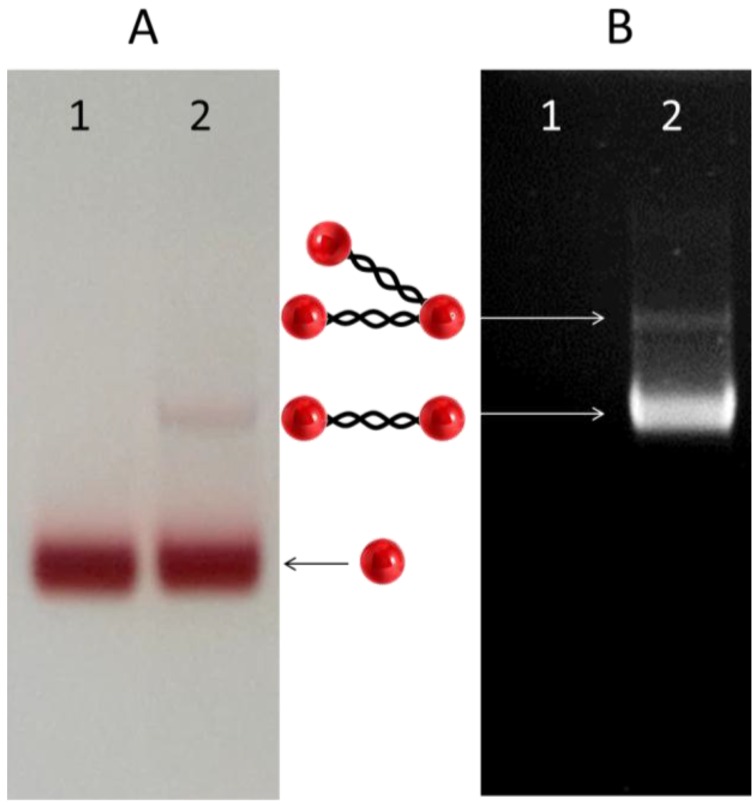
Electrophoresis of DNA–gold nanoparticle (GNPs) conjugates. (**A**) Lane 1: bis-sulfonatophenylphosphine (BSPP)-coated 5 nm GNPs. Lane 2: 450 bp poly(dG)-poly(dC) pre-incubated with the nanoparticles as described in Materials and Methods. The two main wine-colored bands in the image correspond to GNPs (lower band) and the conjugate composed of one DNA molecule and two GNPs. Notice the schematic drawing of the structures between the panels. (**B**) The gel was stained with ethidium bromide for DNA content and visualized with a Bio Imaging System 202D at 302 nm. The minor band above that of the dimer one corresponds to the conjugate bearing two DNA molecules. Notice that the lower band corresponding to GNPs and not containing DNA is invisible in the DNA-stained gel. The electrophoresis was conducted in 2% agarose gel as described in Materials and Methods.

**Figure 2:**
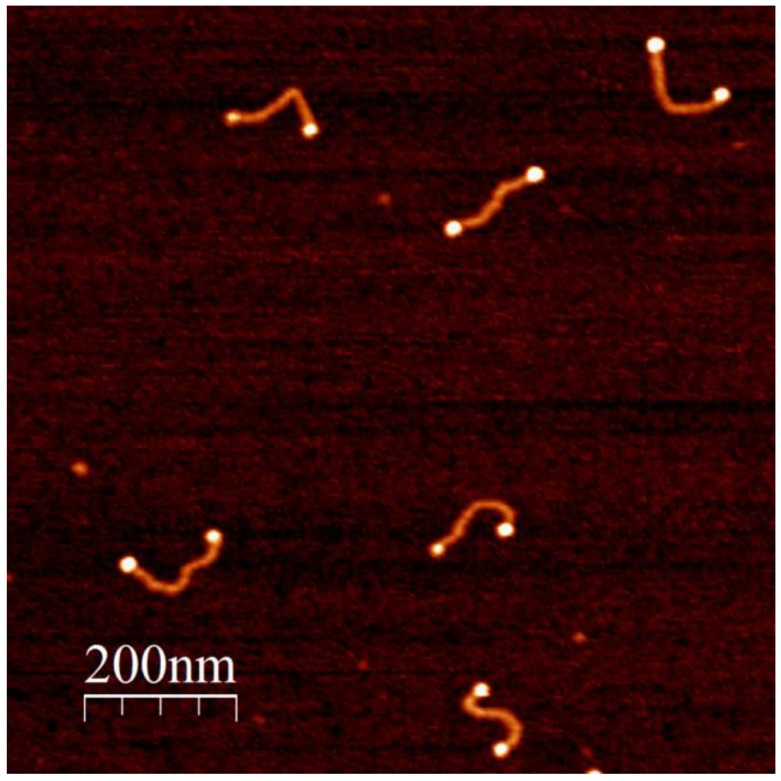
Atomic force microscopy (AFM) image of the DNA-GNP conjugates. The conjugates were prepared, deposited on a mica surface and imaged by AFM in semi-contact (tapping mode) as described in Materials and Methods. Bright spots on the image correspond to the particles and the less bright linear fragments between the spots correspond to the DNA.

Synthesis of SH-poly(dG)-poly(dC)-SH was conducted as described in Materials and Methods and in our earlier publication [16]. Briefly, a double-stranded (ds) template primer, S-S-5'-(dG)_12_-(dC)_12_-5'-S-S, composed of 12 GC base pairs (bp) and containing disulfide groups at both 5' ends of the DNA, was extended by Klenow exo- fragments of Polymerase I in the presence of dGTP and dCTP. The length of the polymer obtained by this method can be varied from tens to tens of thousands of base pairs. In this study we used 450 bp poly(dG)-poly(dC) for conjugation with the nanoparticles. The synthesized DNA was treated with dithiothreitol (DTT) in order to convert the disulfide residues at its ends to thiols. The DNA was completely separated from DTT by size exclusion chromatography (see Materials and Methods) and conjugated with bis-sulfonatophenylphosphine (BSPP)-coated 5 nm GNPs. The particles were synthesized and coated with BSPP as described in Materials and Methods. Treatment with BSPP is required to avoid non-specific binding of the particles to the DNA’s backbone. The DNA was incubated with 10 molar excess of GNPs for 16–20 h under ambient conditions. It is essential to keep the nanoparticle-DNA ratio high. Incubation of the particles in the presence of relatively low concentrations of nanoparticles (the GNP to DNA ratio < 1) yields conjugates bearing several DNA molecules per nanoparticle. At equimolar concentrations, the incubation yields high molecular weight structures composed of a large number of nanoparticles and DNA molecules. In the experimental conditions used here, the majority of conjugates are composed of two GNPs attached to the ends of a DNA molecule. As seen in Figure 1, the lower band in the gel (see panel A) corresponds to GNPs not connected to the DNA, the one above it to the dimer and the low intensity band (see panel B) above that of the dimer to the trimer. In order to separate the conjugates from the excess of GNPs, the incubation was chromatographed on a Sepharose CL 6B column. The column efficiently separates the conjugates from the particles (see Figure S1), though it (in contrast to the electrophoresis) is incapable of separating different DNA-GNP conjugates. The conjugates eluted from the column were collected, deposited on mica and imaged by AFM (see Figure 2). The majority of the structures seen in the AFM image are dumbbell-shaped. The average contour length of the DNA in the conjugates is approximately equal to 150 nm. This length corresponds nicely with 450 bp DNA. 

### 2.2. Enlargement of GNPs in the Conjugates

**Figure 3:**
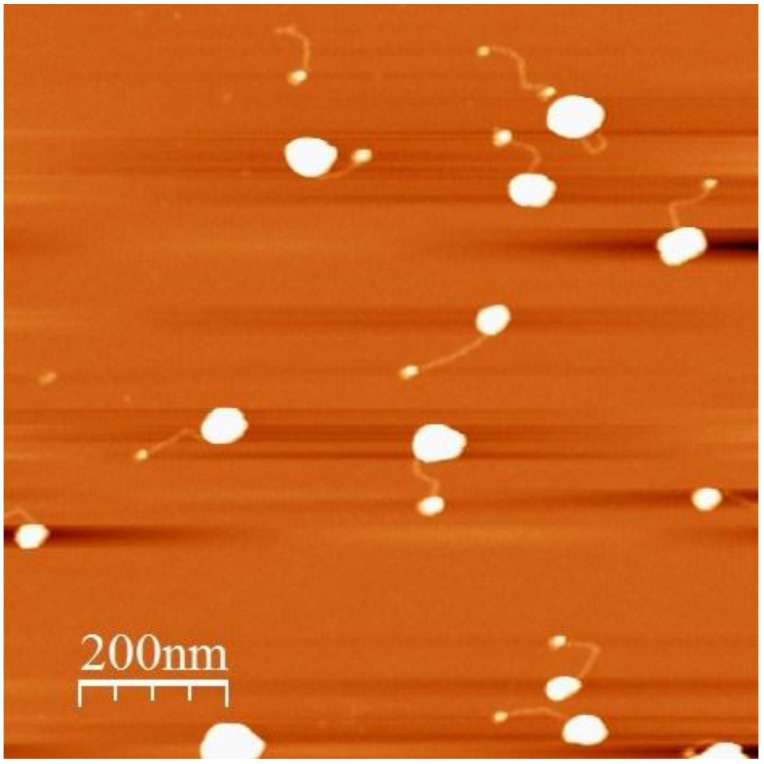
AFM image of DNA-GNP conjugates after treatment of the mica surface with a mixture of HAuCl_4_ and ascorbic acid. The conjugates were deposited on mica as in Figure 2 and treated with a mixture of 20 µM HAuCl_4_ and 2 mM ascorbic acid for 20 s using the “gold enhancement setup” (see Figure S2). The structures were imaged as described in Materials and Methods.

The size of the nanoparticles in the conjugates deposited on the substrate was increased by treatment of the surface with a mixture of ascorbic acid (asc) and gold ions (HAuCl_4_). The reduction of gold ions on the surface of the nanoparticle leads to its continuous growth. If a certain volume of asc-HAuCl_4_ mixture was just poured on the substrate, huge aggregates composed of a large number of nanoparticles would be formed in 1–2 min. These aggregates almost completely cover the mica surface, making AFM scanning impossible. In order to avoid the formation of the aggregates we employed a nanoparticle enhancement setup illustrated by the drawing in Figure S2. The ascorbic acid and HAuCl_4_ solutions were pumped by peristaltic pumps to a mixing chamber, mixed there, and poured dropwise on the substrate (with deposited conjugates) right after the mixing. Figure 3 shows that during this treatment, the particles in the conjugates grew. The treatment, however, leads to unequal enlargement of GNPs; some of the particles reached a height of 20–30 nm, while others did not grow at all. 

**Figure 4:**
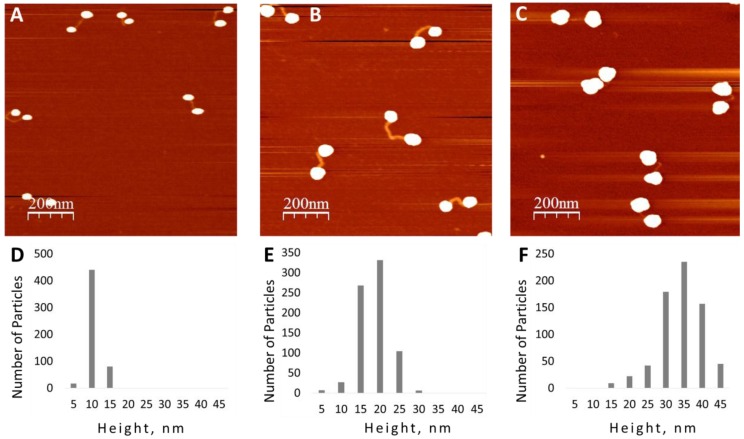
AFM image and statistic height analysis of GNPs in the conjugates. The molecules were deposited on mica as in Figure 2. The substrate was treated with 0.4 mM HAuCl_4_ for 2 min, rinsed with ultra-pure water, and subsequently treated with a mixture of 2 mM ascorbic acid and 1 (**A**), 5 (**B**) and 20 µM (**C**) HAuCl_4_ for 20 s using the “gold enhancement setup” (see Figure S2). Finally the substrate was rinsed with ultra-pure water, dried with nitrogen gas and imaged by AFM as described in Materials and Methods. (**D**–**F**) present statistical height analysis of the nanoparticles in (**A**–**C**), respectively. The average height of the particles is equal to: 10.2 ± 2.7, 18.5 ± 3.8 and 29.3 ± 6 nm for (**A**–**C**), respectively.

Conjugates composed of a big and a small GNPs are clearly seen in Figure 3. The unequal growth of the nanoparticles can be attributed to different coatings of the nanoparticle surface by BSPP. BSPP molecules can dissociate from the nanoparticles, leaving some of them not completely protected by the ligand. These particles should be more susceptible to the enhancement compared to those completely protected by BSPP. In order to reduce the amount of BSPP moieties on the GNP surface and, by this means, to increase its reactivity, we have treated the substrate with deposited conjugates by HAuCl_4_ prior to the extension procedure. Gold ions are characterized by very high affinity to phosphins [17] and treatment with gold ions can remove BSPP from the surface of the nanoparticle. Indeed, the treatment considerably affected the enlargement process (see Figure 4). As seen in Figure 4, all particles in the conjugates have approximately the same size. We have shown that the average height of GNPs depends on the HAuCl_4_ concentration during the enlargement procedure. As seen in Figure 4, treatment of the surface with a mixture of 2 mM ascorbic acid and 1, 5 and 15 µM HAuCl_4_ yields 10.2 ± 1.8, 18.5 ± 3.8 and 29.3 ± 6 nm GNPs, respectively.

Self-assembly properties of DNA make this biopolymer an attractive candidate for the construction of two- and three-dimensional architectures for nanotechnology and nanoelectronics. The prerequisite for creating DNA-based nanoelectronic devices is that DNA molecules can efficiently conduct electrical current. The electronic properties of DNA are, however, very controversial [18,19,20,21,22]. This is mainly due to challenges related to measurement of DNA conductivity and, in particular, in establishing electrical contact between the DNA and the electrodes. The dumbbell-shaped DNA-GNP conjugates reported here can provide a solution for these challenges. The nanoparticles attached to the DNA ends should promote electron transfer between the metal electrode and the organic polymer. The ability to enlarge the particles makes it possible to reduce the edge-to-edge interparticle distance in the dumbbell-shaped conjugate and, as a consequence, to reduce the length of the DNA “bridge” connecting the nanoparticles (see Figure S3). We believe that good electrical contact between the DNA and electrodes, together with the ability to “tune” the length of the nucleic acid fragment bridging the two particles, will allow reliable measurements of charge transport through the DNA. 

## 3. Materials and Methods 

### 3.1. Materials 

Unless otherwise stated, reagents were obtained from Sigma-Aldrich (St. Louis, MO, USA) and were used without further purification. Klenow fragment exonuclease minus of DNA polymerase I from *E. coli* lacking the 3′-, 5′-exonuclease activity (Klenow exo-) was purchased from Epicenter Biotechnologies (Madison, WI, USA) and puc 19 from Thermo Fisher Scientific (Waltham, MA, USA).

### 3.2. Oligonucleotide Purification

Oligonucleotides used in this study, S-S-5'-(dG)_12_ and S-S-5'-(dC)_12_, were purchased from SBS Genetech (Beijing, China). The disulfide groups were linked to the terminal 5' ends of the oligonucleotides via six-carbon linker. Each of the above oligonucleotides (~1 mg) was dissolved in 0.1–0.2 mL of 0.1 M LiOH. The oligonucleotide solutions were centrifuged on a table Eppendorf centrifuge (Hamburg, Germany) at 14,000 rpm for 2 min, in order to get rid of insoluble material that may be present in the oligonucleotide samples. The oligonucleotides were mixed at equimolar concentrations. Concentrations of S-S-5'-(dG)_12_ and S-S-5'-(dC)_12_ were calculated using extinction coefficients at 260 nm of 141.6 mM^−1^·cm^−1^ and 88.8 mM^−1^·cm^−1^, respectively. The oligonucleotide mixture was dialyzed against 20 mM tris-acetate buffer (pH 7.5) for 15–20 h. The dialyzed oligonucleotides were stored at 4 °C. 

### 3.3. Enzymatic Synthesis of DNA

Synthesis of 450 bp [S-S-5'-poly(dG)-3']-[3'-poly-(dC)-5'-S-S], the ds nucleic acid containing disulfide groups at both 5' ends of the molecule was performed as previously described [16,23] using Klenow exo- of DNA Polymerase I. A standard reaction mixture contained: 60 mM K-Pi (pH 7.5), 3.2 mM MgCl_2_, 5 mM DTT, 1 mM dCTP, 1 mM dGTP, 0.025 U/µL Klenow exo- and 2 µM of the template-primer. The enzymatic reaction was conducted for ~20 h at 25 °C. The synthesized DNA molecules were separated from nucleotides and other components of the reaction mixture (including very long DNA molecules that may be present in the mixture in minor quantities) on a TSK-gel G-DNA-PW HPLC column (7.8 × 300 mm) from TosoHaas (Tokyo, Japan). The elution was with 20 mM tris-acetate (pH 7.5) for 30 min at a flow rate of 0.5 mL·min^−1^. The chromatography was conducted on an Agilent 1100 HPLC system (Thermo Electron Corporation, Beverly, MA, USA) with a photodiode array detector. Elution of the DNA was followed at 260 nm. 

### 3.4. Synthesis of GNPs 

GNPs (5 nm in diameter) were prepared by the modified citrate reduction method [24]. To an Erlenmeyer flask with 177 mL of deionized/filtered water, 60.9 µL of 0.85 M HAuCl_4_, and 1.8 mL of 50 mM sodium-citrate were added under constant stirring. The solution was incubated for 10 min at 25 °C. Then 0.45 mL of freshly prepared 0.5 M NaBH_4_ was rapidly added to the mixture under vigorously stirring. The solution almost immediately turned wine red, indicating formation of GNPs. The nanoparticle solution was left for at least 12 h at room temperature in the dark. The particles were coated with BSPP (Strem Chemicals, Newburyport, MA, USA) as follows: 180 µL of 0.1 M BSPP were added under vigorous stirring to 180 mL of the GNP solution and left at room temperature in the dark for ~20 h. The BSPP-coated particles were concentrated on Millipore 30k Centriprep units at 1000 g. The volume of resulting GNPs should be approximately 1 mL. The concentrated nanoparticles were separated from the excess of BSPP by chromatography on a Miditrap G-25 Sephadex column (GE Healthcare, Little Chalfont, UK) equilibrated with 20 mM tris-acetate buffer (pH 7.5). The nanoparticles were collected and used for preparation of DNA-GNP conjugates. Concentration of the particles was calculated using an extinction coefficient (ε) of 9.3 × 10^6^ M^−1^·cm^−1^ at 517 nm.

### 3.5. Synthesis of DNA-GNP Conjugates

In order to attach the particles to the DNA the disulfide groups on both ends of the synthesized polymer, [S-S-5'-poly(dG)]-[poly-(dC)-5'-S-S], should be converted to thiols. To do so, the DNA prepared as described in section 3.3 was treated with 100 mM DTT for 30 min at room temperature. The DNA was separated from the excess of DTT by chromatography on a Miditrap G-25 Sephadex column (GE Healthcare, Little Chalfont, UK). The column was equilibrated with 20 mM K-Pi (pH 6.5). The buffer was bubbled with argon gas for more than an hour to completely remove oxygen. The void volume fraction was collected and mixed with the nanoparticles prepared as described in 3.4. Concentrations of the DNA and GNPs in the incubation were 0.1 and 10 µM respectively. 50 mM LiCl was added and the incubation was bubbled with argon gas for 2 h. The tube was sealed and the incubation was left at room temperature in the dark for ~70 h. The incubation (~1 mL) was then chromatographed on a Sepharose 2B-CL column (1.6 × 10 cm) equilibrated with 20 mM tris-acetate (pH 7.5). The elution was with isocratic gradient at a flow rate of 1 mL/min. The DNA-GNP conjugates were eluted close to the void volume while GNPs were eluted in about one-half column volume (see Figure S1). The conjugates were collected and imaged by AFM. 

### 3.6. Atomic Force Microscopy

AFM was performed on molecules adsorbed on muscovite mica. 100 µL of DNA-GNP conjugates (absorbance is ~5 mOD at 260 nm) in 1 mM Mg-acetate was deposited on a freshly cleaved 1 × 1 cm mica plate; 5 min after, the surface was rinsed with ultra-pure distilled water and dried by blowing nitrogen gas. AFM imaging was performed on a Solver PRO AFM system (NT-MDT, Russia), in a semi-contact (tapping) mode, using Si-gold-coated cantilevers (NT-MDT, Zelenograd, Russia) with resonance frequency of 80–110 kHz. The images were “flattened” (each line of the image was fitted to a second-order polynomial, and the polynomial was then subtracted from the image line) by the Nova image processing software (NT-MDT, Zelenograd, Russia). The images were analyzed and visualized using a Nanotec Electronica S.L (Madrid, Spain) WSxM imaging software [25].

## 4. Conclusions

We synthesized dumbbell-shaped structures in which the two 5 nm GNPs are connected by a 450 bp poly(dG)-poly(dC) DNA molecule. The particles in the nanodumbbells were enlarged to approximately 10 nm, 20 nm and 30 nm in diameter. The enlargement method that we developed might be applicable to DNA-nanoparticle structures, such as complexes of DNA origami with nanoparticles.

## Supplementary Materials

The supplementary materials are available online at http://www.mdpi.com/2079-4991/6/9/167/s1.

## Abbreviations

DTT—dithiothreitol; dGTP—2′-deoxyguanosine 5′-triphosphate; dCTP—2′-deoxycytidine 5′-triphosphate; GNPs—gold nanoparticles; AFM—Atomic Force Microscopy; poly(dG)-poly(dC)—a double-stranded DNA polymer consisting of poly(dC) and poly(dG) homopolymer chains; BSPP—Bis(p-sulfonatophenyl)phenylphosphine; asc—ascorbic acid.

## References

[1] Mirkin C.A., Letsinger R.L., Mucic R.C., Storhoff J.J. (1996). A DNA-based method for rationally assembling nanoparticles into macroscopic materials. Nature.

[2] Alivisatos A.P., Johnsson K.P., Peng X., Wilson T.E., Loweth C.J., Bruchez M.P., Schultz P.G. (1996). Organization of ‘nanocrystal molecules’ using DNA. Nature.

[3] Loweth C.J., Caldwell W.B., Peng X., Alivisatos A.P., Schultz P.G. (1999). DNA-Based Assembly of Gold Nanocrystals. Angew. Chem. Int. Ed..

[4] Claridge S.A., Alivisatos A.P. (2009). Pyramidal Chiral Groupings of Gold Nanocrystals Assembled Using DNA Scaffolds. J. Am. Chem. Soc..

[5] Fan J.A., He Y., Bao K., Wu C., Bao J., Schade N.B., Manoharan V.N., Shvets G., Nordler P., Liu D.R., Capasso F. (2011). DNA-Enabled Self-Assembly of Plasmonic Nanoclusters. Nano Lett..

[6] Aldaye F.A., Sleiman H.F. (2006). Sequential self-assembly of a DNA hexagon as a template for the organization of gold nanoparticles. Angew. Chem. Int. Ed..

[7] Aldaye F.A., Sleiman H.F. (2007). Dynamic DNA templates for discrete gold nanoparticle assemblies: Control of geometry; modularity; write/erase structural switching. J. Am. Chem. Soc..

[8] Zheng J., Constantinou P.E., Micheel C., Alivisatos A.P., Kiehl R.A., Seeman N.C. (2006). Two-dimensional nanoparticle arrays show the organizational power of robust DNA motifs. Nano Lett..

[9] Schreiber R., Santiago I., Ardavan A., Turberfield A.J. (2016). Ordering Gold Nanoparticles with DNA Origami Nanoflowers. ACS Nano.

[10] Gür F.N., Schwarz F.W., Ye J., Diez S., Schmidt T.L. (2016). Toward Self-Assembled Plasmonic Devices: High-Yield Arrangement of Gold Nanoparticles on DNA Origami Templates. ACS Nano.

[11] Borovok N., Gillon E., Kotlyar A. (2012). Synthesis Assembly of Conjugates Bearing Specific Numbers of DNA Strs per Gold Nanoparticle. Bioconjug. Chem..

[12] Lim D.K., Jeon K.S., Kim H.M., Nam J.M., Suh Y.D. (2010). Nanogap-engineerable Raman-active nanodumbbells for single-molecule detection. Nat. Mater..

[13] Zikich D., Borovok N., Molotsky T., Kotlyar A. (2010). Synthesis AFM characterization of poly(dG)-poly(dC)-gold nanoparticle conjugates. Bioconjug. Chem..

[14] Yang X., Wang X.-B., Vorpagel E.R., Wang L.S. (2004). Direct experimental observation of the low ionization potentials of guanine in free oligonucleotides by using photoelectron spectroscopy. Proc. Natl. Acad. Sci. USA.

[15] Hennig D., Starikov E.B., Archilla J.F.R., Palmero F. (2004). Charge transport in poly(dG)-poly(dC) poly(dA)-poly(dT) DNA polymers. J. Biol. Phys..

[16] Kotlyar A.B., Borovok N., Molotsky T., Fadeev L., Gozin M. (2005). In vitro synthesis of uniform poly(dG)-poly(dC) by Klenow exo- fragment of polymerase I. Nucleic Acids Res..

[17] Assefa Z., Forward J.M., Grant T.A., Staples R.J., Hanson B.E., Mohamed A.A., Fackler J.P. (2003). Three-coordinate; luminescent; water-soluble gold(I) phosphine complexes: Structural characterization photoluminescence properties in aqueous solution. Inorganica Chim. Acta.

[18] De Pablo P.J., Moreno-Herrero F., Colchero J., Gomez Herrero J., Herrero P., Baro A.M., Ordejon P., Soler J.M., Artacho E. (2000). Absence of dc-conductivity in lambda-DNA. Phys. Rev. Lett..

[19] Storm A., Van Noort J., De Vries S., Dekker C. (2001). Insulating behavior for DNA molecules between nanoelectrodes at the 100 nm length scale. Appl. Phys. Lett..

[20] Porath D., Bezryadin A., De Vries S., Dekker C. (2000). Direct measurement of electrical transport through DNA molecules. Nature.

[21] Kasumov A.Y., Kociak M., Gueron S., Reulet B., Volkov V., Klinov D., Bouchiat H. (2001). Proximity-induced superconductivity in DNA. Science.

[22] Eidelshtein G., Kotlyar A., Hashemi M., Gurevich L. (2015). Aligned deposition electrical measurements on single DNA molecules. Nanotechnology.

[23] Kotlyar A. (2011). Synthesis of long DNA-based nanowires. Methods Mol. Biol..

[24] Turkevich J., Stevenson P.C., Hillier J. (1951). A study of the nucleation growth processes in the synthesis of colloidal gold. Discuss. Faraday Soc..

[25] Horcas I., Fernez R., Gomez-Rodriguez J.M., Colchero J., Gomez-Herrero J., Baro A.M. (2007). WSXM: A software for scanning probe microscopy a tool for nanotechnology. Rev. Sci. Instrum..

