# Supplementary material for: Preparation, Characterization and Manipulation of Conjugates between Gold Nanoparticles and DNA
Source: Nanomaterials (Basel). 2016 Sep 8;6(9):167. doi: 10.3390/nano6090167 (PMC5224630; doi:10.3390/nano6090167)
Supplement: Supplementary file 1 [file nanomaterials-06-00167-s001.pdf]

Supplementary Materials: Preparation, Characterization and Manipulation of Conjugates between Gold Nanoparticles and DNA

Gennady Eidelshtein, Moran Fattal, Gavriel Avishai, Benjamin Kempinski, Clelia Giannini and Alexander Kotlyar

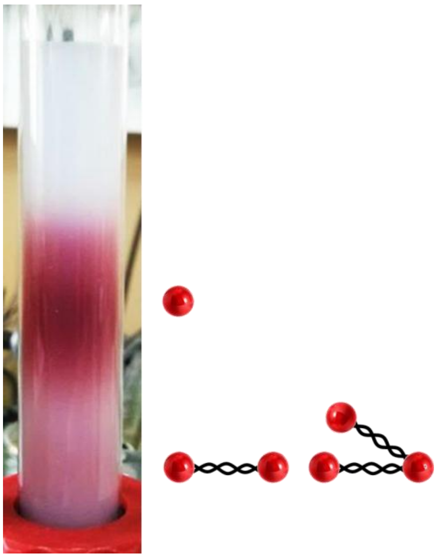

Figure S1. Size-exclusion chromatography of DNA–gold nanoparticle (GNP) conjugates. Conjugates of 450 bp poly(dG)-poly(dC) with 5 nm GNPs prepared as described in Materials and Methods were chromatographed on a size-exclusion Sepharose 2B-CL column (1.6 × 10 cm) equilibrated with 20 mM tris-acetate (pH 7.5). The elution was with isocratic gradient at a flow rate of 1 mL/min. The upper colored area on the column image corresponds to GNPs not connected to DNA, while the lower one (at the very bottom) to DNA-GNP conjugates, composed of NPs and DNA molecules.

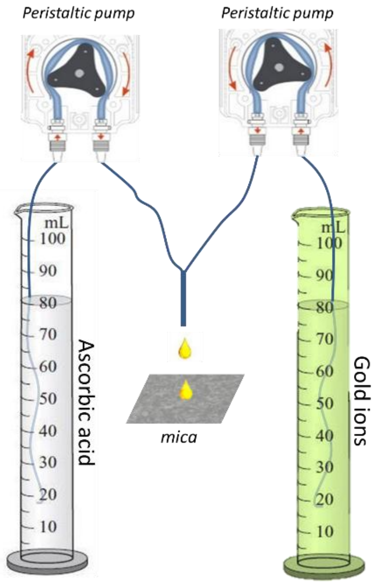

Figure S2. A schematic drawing of the "gold enhancement setup". Two peristaltic pumps are rapidly (flow rate of ~20 mL/min) delivering $HAuCl_4$ and ascorbic acid solutions from cylinders to a mixing chamber where the solutions are mixed together and fall dropwise on the substrate.

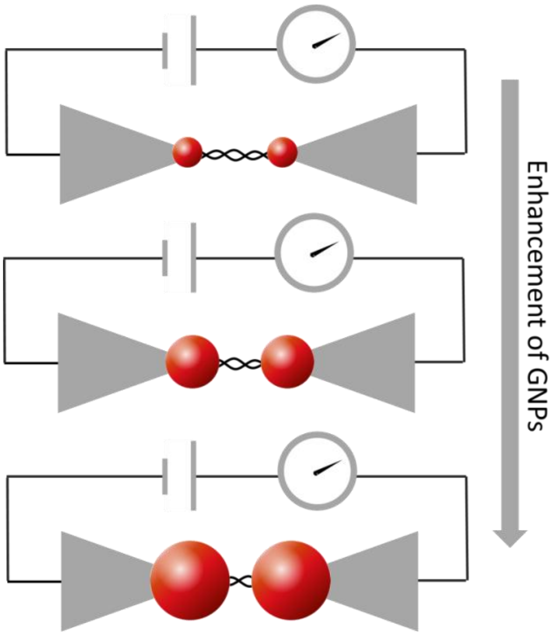

Figure S3. Schematic drawing of electrodes connected by a DNA-GNP conjugate. The electrodes shown on the scheme as grey triangles are separated by 50 nm (a common separation distance for electrodes made by e-beam lithography). The size of GNPs in the conjugate attached to the electrodes is increased during the enhancement process. As a result, the length of the DNA "bridge" separating the two particles is reduced. Thus, by enlarging the nanoparticles in the conjugate, one can "tune" the length of the DNA fragment connecting the electrodes. For example, if the length of the DNA is ~50 nm and the diameter of each of the enlarged nanoparticles in the conjugate is 40 nm, the length of the DNA "bridge" separating the particles is equal to 10 nm.

© 2016 by the authors. Submitted for possible open access publication under the terms and conditions of the Creative Commons Attribution (CC-BY) license (http://creativecommons.org/licenses/by/4.0/).
